# Supplementary material for: Intrinsic factors responsible for brittle versus ductile nature of refractory high-entropy alloys
Source: Nat Commun. 2024 Feb 24;15:1706. doi: 10.1038/s41467-024-45639-8 (PMC10894205; doi:10.1038/s41467-024-45639-8)
Supplement: Supplementary file 1 — Supplementary Information [file 41467_2024_45639_MOESM1_ESM.pdf]

Supplementary Information for

Intrinsic factors responsible for brittle versus ductile nature of refractory high-entropy alloys

Tomohito Tsuru[1,2*], Shu Han[3], Shutaro Matsuura[3], Zhenghao Chen[3], Kyosuke Kishida[3,2*], Ivan Iobzenko[1], Satish I. Rao[4], Christopher Woodward[5] and Easo P. George[6,7*], Haruyuki Inui[3,2*]

[1] Nuclear Science and Engineering Center, Japan Atomic Energy Agency, 2-4 Shirakata, Tokai-mura, Ibaraki 319-1195, Japan

[2] Center for Elements Strategy Initiative for Structural Materials (ESISM), Kyoto University, Sakyo-ku, Kyoto 606-8501, Japan

[3] Department of Materials Science and Engineering, Kyoto University, Sakyo-ku, Kyoto 606-8501, Japan

[4] Department of Mechanical Engineering, Johns Hopkins University, Baltimore, MD 21218, USA

[5] Materials and Manufacturing Directorate, Air Force Research Laboratory (retired), Wright Patterson Air Force Base, Dayton, OH 45433-7817, USA

[6] Department of Materials Science and Engineering, University of Tennessee, Knoxville, TN 37996, USA

[7] Institute for Materials, Ruhr University Bochum, 44801 Bochum, Germany

Correspondence to:

tsuru.tomohito@jaea.go.jp;

kishida.kyosuke.6w@kyoto-u.ac.jp;

egeorge@utk.edu;

inui.haruyuki.3z@kyoto-u.ac.jp

Supplementary Tables

Supplementary Table 1 | Parameters used to fit experimental yield stress-temperature curves of VNbMoTaW and TiZrNbHfTa to the equation:

$\sigma(T)=\left\{\sigma_{\mu}+\sigma_{\text{th}}(0)\left[1-(T/T_{\text{TA}})^{1/q}\right]^{1/p}\right\}$, where $\sigma(T)$ is the yield stress at absolute temperature T, σ_{μ} is the athermal stress at the critical or athermal temperature T_{TA}, $\sigma_{\text{th}}(0)$ is the extrapolated yield stress at 0 K, and p and q are fitting constants.

	σ_{μ} (MPa)	$\sigma_{\text{th}}(0)$ (MPa)	T_{TA} (K)	q	p
VNbMoTaW	830	2650	973	1.10	0.59
TiZrNbHfTa	798	3200	673	1.64	0.54

Supplementary Table 2 | Solute-screw dislocation interaction energies for various solutes in TiZrNbHfTa.

**Solute**	**Interaction energy (eV)**
Ti	-0.045
Zr	-0.059
Nb	0.048
Hf	-0.046
Ta	0.078

Supplementary Table 3 | Solute-screw dislocation interaction energies for various solutes in VNbMoTaW.

**Solute**	**Interaction energy (eV)**
V	-0.072
Nb	-0.064
Mo	0.041
Ta	-0.027
W	0.091

Supplementary Table 4 | Square root of the MSAD values of each constitutive element, and the average of all elements, normalized by the Burgers vector in VNbMoTaW and TiZrNbHfTa.

**VNbMoTaW**	
**Element**	$\sqrt{\mathbf{MSAD}}$ / $\boldsymbol{b}$
V	0.0276
Nb	0.0235
Mo	0.0209
Ta	0.0253
W	0.0205
Ave	0.0237

**TiZrNbHfTa**	
**Element**	$\sqrt{\mathbf{MSAD}}$ / $\boldsymbol{b}$
Ti	0.0914
Zr	0.0666
Nb	0.0558
Hf	0.0661
Ta	0.0633
Ave	0.0698

Supplementary Table 5 | Frequency distribution of dislocation dipole energy in VNbMoTaW and TiZrNbHfTa.

VNbMoTaW	
**Energy**	**Frequency**
11.50	1
11.75	4
12.00	9
12.25	14
12.50	21
12.75	28
13.00	23
13.25	14
13.50	7
13.75	5
14.00	8
14.25	1
14.50	0

TiZrNbHfTa	
**Energy**	**Frequency**
3.00	0
3.25	0
3.50	5
3.75	7
4.00	17
4.25	32
4.50	31
4.75	25
5.00	11
5.25	5
5.50	2
5.75	0
6.00	0

Supplementary Table 6 | Vacancy and self-interstitial formation energies of the relevant BCC elements [1,2].

Element	Vacancy formation energy (eV)	Self-int formation energy (eV)
Nb	2.99	5.25
Ta	3.14	5.83
Mo	2.96	7.42
W	3.56	9.55
V	2.51	3.31
Ti	1.55	2.33
Hf	2.0	3.0
Zr	1.7	2.55

Supplementary Figures

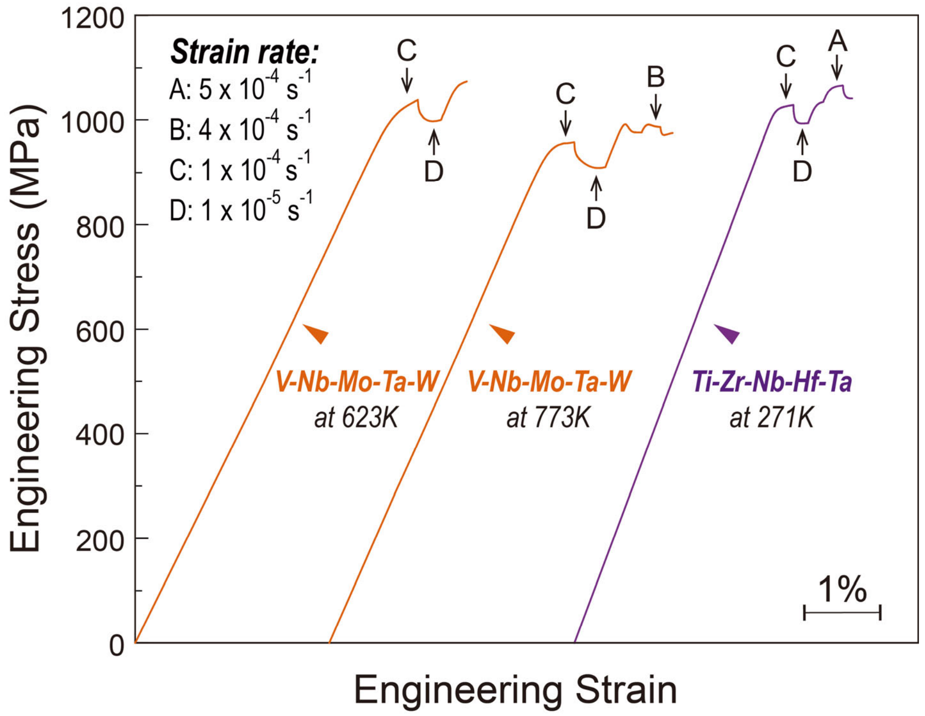

Supplementary Fig. 1 | Selected compressive stress-strain curves of polycrystalline VNbMoTaW and TiZrNbHfTa for strain rate jumps between 1 × 10^{-5} s^{-1} and 5 × 10^{-4} s^{-1}. The activation enthalpy for deformation (H*) was evaluated from results of strain-rate jump tests with the equation: $H^{*} = -TkT\ln\left(\dot{\varepsilon}_2/\dot{\varepsilon}_1\right)/\left(\sigma_2-\sigma_1\right)\partial\sigma/\partial T$, where σ_i is flow strength when tested at a strain rate of $\dot{\varepsilon}_i$, and k is the Boltzmann constant.

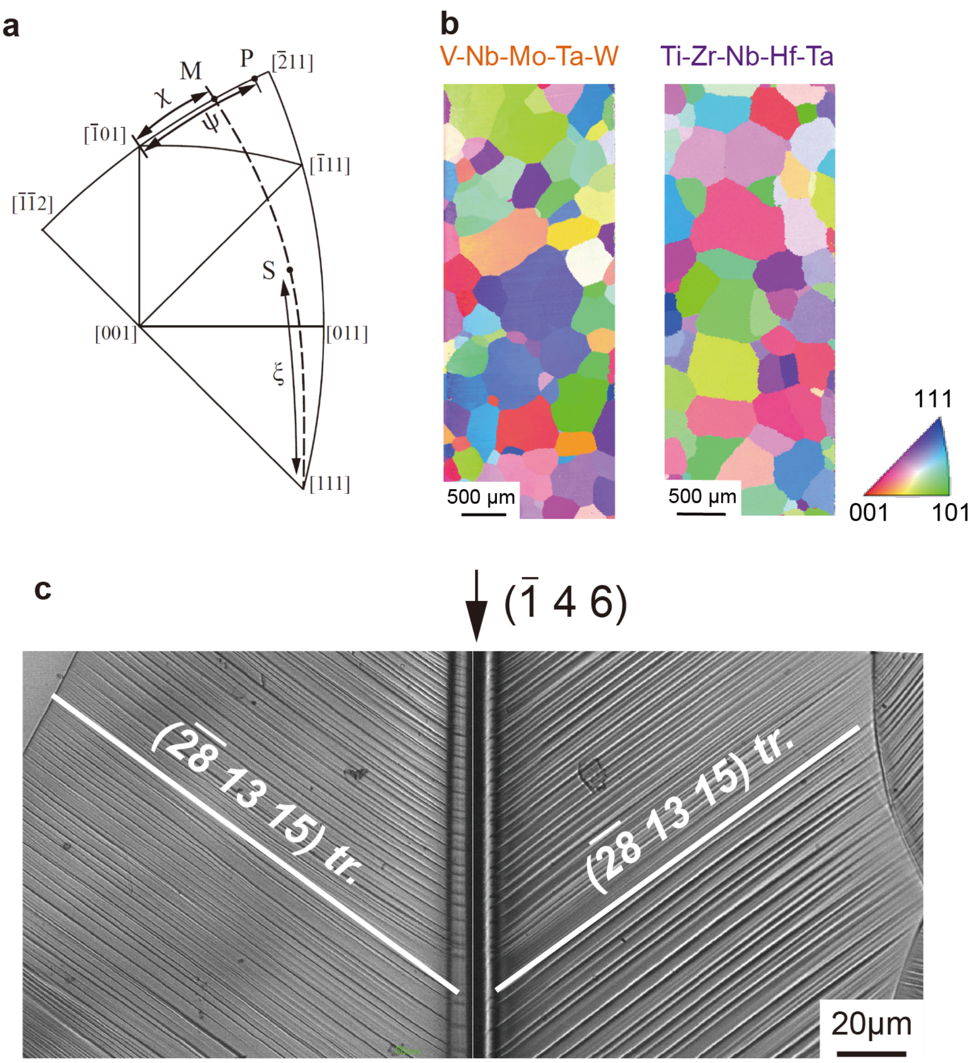

Supplementary Fig. 2 | a, Stereographic projection showing slip behavior in BCC metals, where S corresponds to the loading axis, M is the maximum resolved shear stress plane, P is the observed slip plane, χ is the angle between primary slip plane and M, and Ψ is the angle between primary slip plane and P. **b,** EBSD orientation maps of the specimens used for compression test. **c,** Light microscope images of typical slip traces observed on adjacent specimen surfaces after compression. The active slip plane determined by slip trace analysis is indicated.

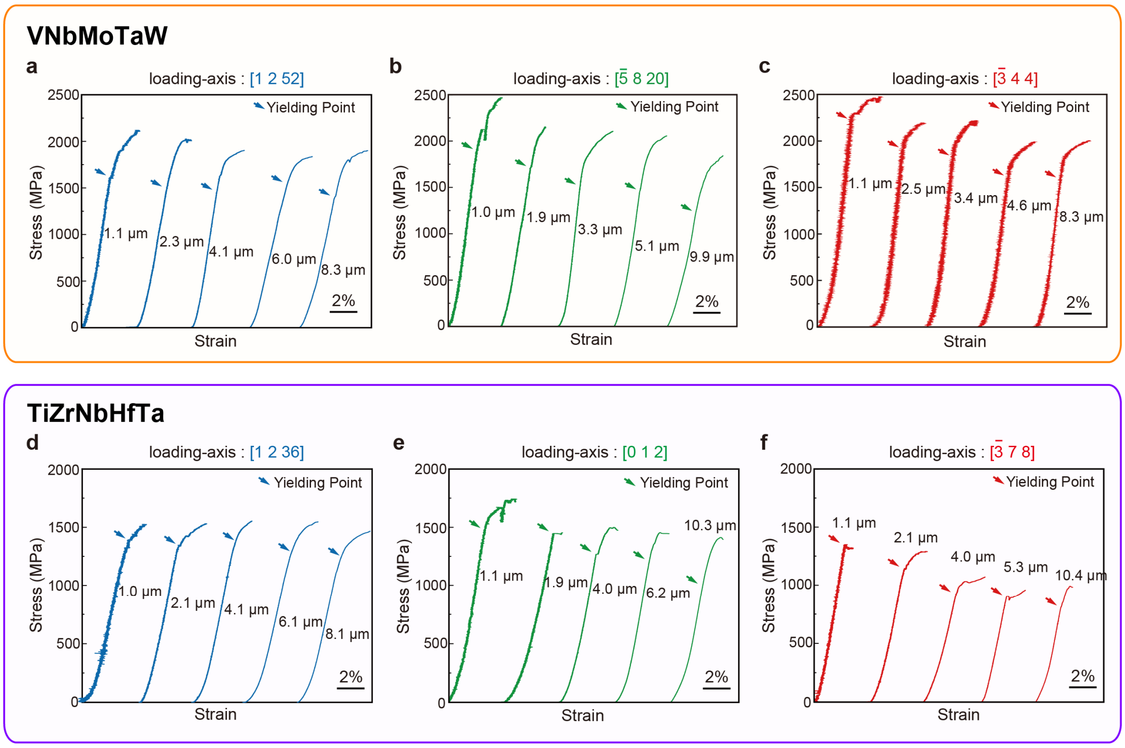

Supplementary Fig. 3 | Selected stress-strain curves from micropillar compression tests on single crystals of **a-c,** VNbMoTaW and **d-f,** TiZrNbHfTa**,** with the orientations of the loading axis labeled above each figure.

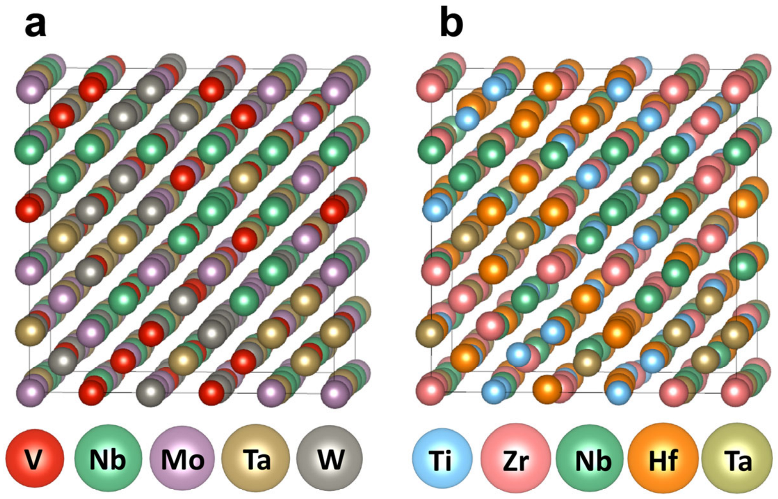

c

	VNbMoTaW		TiZrNbHfTa
r_V	1.34	r_{Ti}	1.47
r_{Nb}	1.46	r_{Zr}	1.60
r_{Mo}	1.39	r_{Nb}	1.46
r_{Ta}	1.46	r_{Hf}	1.59
r_W	1.39	r_{Ta}	1.46
δ	3.282	δ	4.267

Supplementary Fig. 4 | Typical examples of atomic configuration after full structural relaxation of SQS model with random configuration in **a**, VNbMoTaW and **b**, TiZrNbHfTa. **c**, Atomic radii and δ parameter defined as $\delta = \sqrt{\sum_{i=1}^{n} c_i \left(1 - r_i / \bar{r}\right)^2} \times 100$ (c_i and r_i are the atomic concentration and atomic radius of element "i", and $\bar{r}$ is the average of the atomic radii) for VNbMoTaW and TiZrNbHfTa.

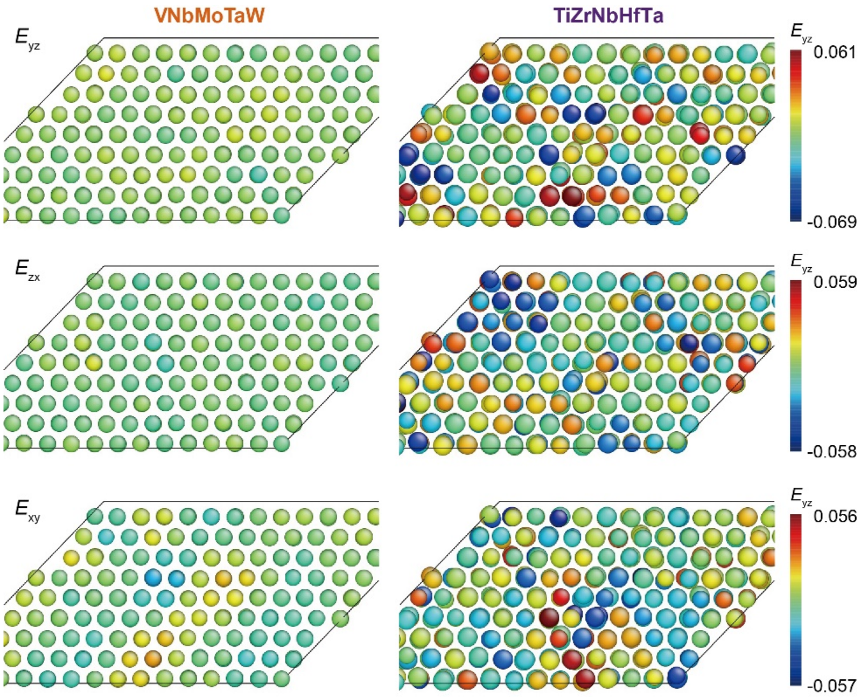

Supplementary Fig. 5 | Local atomic strain based on Green-Lagrange strain tensor. The shear strain components, E_{yz}, E_{zx}, and E_{xy} are visualized for VNbMoTaW and TiZrNbHfTa. These shear strain components contribute to the increase in the energy barrier to the screw dislocation motion resulting in high modulus-normalized yield strength in TiZrNbHfTa.

Supplementary Discussion

Fracture is a "weakest link" phenomenon. As such, the various intrinsic and extrinsic factors that can lead to premature (brittle) fracture must all be suppressed (or overcome) before significant ductility can be achieved (see [3] for a recent overview of this). Intrinsic factors include, for example, sufficient mobile dislocation density, ease of dislocation motion and multiplication, and sustained work hardening. Extrinsic factors include, for example, surface cracks, flaws introduced during processing and machining, and environmental effects. Extrinsic factors are less likely to cause embrittlement if the material is intrinsically ductile; therefore, materials scientists are motivated to understand and overcome intrinsic factors that lead to brittleness.

Sticking to the alloys at hand, TiZrHfNbTa displays plastic elongations of ~8% with limited work hardening [4] and a remarkably high fracture toughness of 210 MPa $m^{1/2}$ reported in 3-point bending [5]. In contrast, VNbMoTaW displays zero tensile elongation (and less than 3% ductility even in compression, ref. [6]) suggesting that its resistance to crack growth (fracture toughness) is likely to be very low indeed (although no results of fracture toughness tests have been reported to date). It stands to reason, therefore, that intrinsic factors are largely responsible for these dramatic differences.

Our goal in selecting these two model alloys for investigation was to understand at least some of the responsible intrinsic factors. As summarized before, we identified several distinctive differences in the properties of these alloys, including the following. TiZrHfNbTa has a spread dislocation core while that of VNbMoTaW is compact; its shear modulus is also lower than that of VNbMoTaW. The lattice distortion (both near the dislocation core and far away) was greater in TiZrHfNbTa than in VNbMoTaW. As a result, the dislocation core energy is lower in TiZrHfNbTa, making it easier to nucleate dislocations. Our preliminary results indicate that the valleys in the energy landscape of TiZrHfNbTa are shallower, potentially making dislocation motion (and not just dislocation nucleation) easier. We were able to link all these effects to the presence of HCP elements in TiZrHfNbTa (VNbMoTaW consists of only BCC elements).

Some of the factors identified in the present study may be rationalized by invoking a Rice-Thomson type view of fracture [7]. In their model, a sharp cleavage crack is blunted and prevented from advancing if dislocations are spontaneously emitted from the tip of an atomically sharp crack. Blunting (ductile behavior) occurs more easily when the dislocation core is wide, the shear modulus (G) is low, and the surface energy (γ) is high. The first two of these factors favors TiZrHfNbTa. However, the third factor, surface energy, should favor VNbMoTaW because it generally scales with the melting point (by the same token, the ideal (Griffith) fracture strength (in cleavage) of VNbMoTaW should also be higher). Another criterion that is often invoked to "predict" whether a material will be brittle or ductile is the empirical Pugh [8] ratio, G/K, where K is the bulk modulus. As has been noted before [9], K has been empirically observed to correlate with γ, which is related to the ideal work of fracture, and G is related to plastic flow. Thus, the Rice-Thomson criterion (Gb/γ), where b is the dislocation Burgers vector, is analogous to the Pugh ratio, when metals with the same crystal structure and similar values of b are compared.

Notwithstanding the above, it has been argued [10] that fracture is not a simple either/or phenomenon as envisaged in the Rice-Thomson model; rather, plastic deformation and crack propagation often occur simultaneously. Consequently, relatively small changes in the surface (or interface energy in the case of, say, intergranular fracture) on the one hand, and shear modulus or ease of dislocation nucleation and motion on the other hand can have nonlinear multiplier effects on the associated plastic work of fracture, thereby altering whether failure occurs in a ductile or brittle manner.

Given the complexity of fracture, our goal here was rather more modest than to develop a comprehensive understanding of all the factors responsible for brittle vs. ductile fracture. And in that endeavor, we believe we have succeeded. We also believe our new understanding can be applied in the development of new RHEAs that have better combinations of strength and ductility than those currently known.

Supplementary References

1. Ghafarollahi, A. Strengthening mechanisms in dilute and high-entropy alloys. PhD Thesis, EPFL, 2022.
2. Derlet, P. M., Nguyen-Manh, D. & Dudarev, S. I. Multiscale modeling of crowdion and vacancy defects in body-centered-cubic transition metals. Phys. Rev. B 76 (2007) 054107.
3. George, E. P. & Ritchie, R. O. High-entropy materials. *MRS Bull.* **47**, 145–150 (2022).
4. Senkov, O. N., Wilks, G. B., Scott, J. M. & Miracle, D. B. Mechanical properties of Nb25Mo25Ta25W25 and V20Nb20Mo20Ta20W20 refractory high entropy alloys. *Intermetallics* **19**, 698–706 (2011).
5. Fan, X. J., Qu, R. T. & Zhang, Z. F. Remarkably high fracture toughness of HfNbTaTiZr refractory high-entropy alloy. *J. Mater. Sci. Tech.* **123**, 70–77 (2022).
6. Senkov, O. N. & Semiatin, S. L. Microstructure and properties of a refractory high-entropy alloy after cold working. *J. Alloys Compd.* **649**, 1110–1123 (2015).
7. Rice, J. R. & Thomson, R. Ductile versus brittle behaviour of crystals. *Philos. Mag. A* **29**, 73–97 (1974).
8. Pugh, S. F. XCII. Relations between the elastic moduli and plastic properties of polycrystalline pure metals. *The London, Edinburgh, and Dublin Philos. Mag. and Journal of Science*, **45** (367), 823-843 (1954).
9. George, E. P., Horton, J. A., Porter, W. D., Schneibel, J. A. Brittle cleavage of $L1_2$ trialuminides. J. Mater. Res. 5, 1639-1648 (1990).
10. Jokl, M. L., Vitek, V. & McMahon Jr, C. J. A microscopic theory of brittle fracture in deformable solids: A relation between ideal work to fracture and plastic work. *Acta Metall.* **28**, 1479–1488 (1980).
